# An Overview of Environmental Risk Factors for Food Allergy

**DOI:** 10.3390/ijerph19020722

**Published:** 2022-01-10

**Authors:** Rachel L. Peters, Suzanne Mavoa, Jennifer J. Koplin

**Affiliations:** 1 Murdoch Children’s Research Institute, Parkville 3052, Australia; suzanne.mavoa@unimelb.edu.au (S.M.); jennifer.koplin@mcri.edu.au (J.J.K.); 2 Department of Paediatrics, University of Melbourne, Parkville 3052, Australia; 3 Melbourne School of Population and Global Health, University of Melbourne, Parkville 3052, Australia

**Keywords:** food allergy, vitamin D, environmental greenness, air pollution, pollen, biodiversity

## Abstract

IgE-mediated food allergy is an increasing public health concern in many regions around the world. Although genetics play a role in the development of food allergy, the reported increase has occurred largely within a single generation and therefore it is unlikely that this can be accounted for by changes in the human genome. Environmental factors must play a key role. While there is strong evidence to support the early introduction of allergenic solids to prevent food allergy, this is unlikely to be sufficient to prevent all food allergy. The purpose of this review is to summarize the evidence on risk factors for food allergy with a focus the outdoor physical environment. We discuss emerging evidence of mechanisms that could explain a role for vitamin D, air pollution, environmental greenness, and pollen exposure in the development of food allergy. We also describe the recent extension of the dual allergen exposure hypothesis to potentially include the respiratory epithelial barrier in addition to the skin. Few existing studies have examined the relationship between these environmental factors with objective measures of IgE-mediated food allergy and further research in this area is needed. Future research also needs to consider the complex interplay between multiple environmental factors.

## 1. Introduction

IgE-mediated food allergy is a growing public health concern in many regions around the world. Food allergy is characterized by a loss of, or failure to establish, oral tolerance which is the active inhibition of immune responses to usually harmless food antigen. The propensity of the immune system to respond to food antigens in this manner results from dysregulated immune responses and a skewing towards a T helper cell type 2 (Th2) phenotype which is associated with production of immunoglobulin E antibodies (IgE) towards allergens. These mechanisms are reviewed in detail in other sources [1,2].

Estimates of the prevalence of food allergy frequently vary in the published literature. In a meta-analysis, the prevalence of self-reported food allergy ranges from 1.2% to 17% for milk, 0.2% to 7% for egg, 0% to 2% for peanut, significantly higher than estimates based on objective measures such as measures of sensitization (skin prick test and serum specific IgE) which ranged from 2% to 9% for milk, less than 1% to 9% for egg, and less than 1% to 6% for peanut. Estimates based on the gold standard oral food challenge are most robust, which varied from 0% to 3% for milk, 0% to 1.7% for egg, and 1.5% to 1.6% for peanut [3]. Using the gold standard oral food challenge and a population-representative sampling frame, the HealthNuts study in Australia has reported the highest prevalence of challenge confirmed food allergy at 9% for egg and 3% for peanut [4]. The prevalence of challenge-confirmed food allergy is lower in South Africa, at 1.9% to egg, 0.8% to peanut, and 0.1% to cow’s milk [5], and Europe ranging from 0.3% to 1% to milk depending on the country and 0.7% to 2.2% to egg [6,7].

Many factors contribute to variations in food allergy prevalence including age, ethnic and geographical variations, differing awareness of food allergy and access to health care, and infant feeding practices [8]. The prevalence of food allergy has increased over the last several decades, with the rise occurring largely within a single generation. Although genetics play a role, as do complex gene–environment interactions, the high prevalence has occurred faster than can be accounted for by changes in the human genome, and therefore it is highly likely that environmental factors play a key role.

Several hypotheses have been proposed to explain the causes of food allergy including the dual allergen exposure hypothesis, vitamin D hypothesis, and the interrelated microbial exposure and biodiversity hypotheses [2,9,10]. Recently, it has been proposed that changes in the environment as a consequence of industrialization, urbanization, and modern lifestyles impair the integrity of the epithelial barrier of the skin, respiratory and gastro intestinal tracts, predisposing not only to allergic diseases, but also autoimmune and other chronic diseases [11]. This narrative review will provide an overview of risk factors for food allergy with a focus on features of the outdoor physical environment.

## 2. Overview of Risk Factors for Food Allergy Development

### 2.1. Dual Allergen Exposure Hypothesis

The dual allergen exposure hypothesis is widely accepted as a plausible explanation of food allergy development. The premise of this hypothesis is that the damaged epithelial barrier of the skin, as occurs in eczema, facilitates exposure of allergens to the immune system, resulting in sensitization and production of IgE antibodies to the allergen. Food allergy can develop if the infant is not first exposed to the food via the oral route, which would normally induce immunologic tolerance, the active inhibition of immune responses to foods [10].

A substantial body of evidence including randomized controlled trials, supports that early introduction of allergenic solids, specifically peanut and egg, into the infant’s diet reduces the risk of food allergy [12,13,14]. Food allergy prevention guidelines internationally now recommend introduction of these foods into an infant’s diet in the first year of life to reduce the risk of developing food allergy. Despite this, early introduction of allergens is unlikely to prevent all food allergy. Evidence shows that some food allergy develops prior to when infants are developmentally ready to ingest foods. For example, in an RCT of early egg introduction, some infants had evidence of IgE sensitization prior to 4 months of age and reacted to egg powder on their first known exposure to egg [15]. The protective effect of introduction also appears to be allergen specific, as early introduction of peanut for example did not reduce the risk that a child would develop tree nut allergy in an RCT of early peanut introduction [12]. There is currently insufficient evidence to support a protective effect of early introduction of allergenic foods other than peanut and egg. Although it is plausible that similar mechanisms of protection may be present for other foods such as tree nuts, it is also possible that different “windows of opportunity” exist for different foods. One notable example is cow’s milk, with a recent RCT showing a reduction in cow’s milk allergy at age 6 months with daily cow’s milk formula ingestion from 1 to 2 months of age compared with cow’s milk avoidance during this early life period [16].

As well as early oral allergen introduction, studies have attempted to prevent sensitization occurring through the skin. Several studies have shown that application of creams containing peanut oil to eczematous skin, or increased exposure to peanut dust in the home environment among infants with eczema, is associated with an increased risk of peanut sensitization and allergy [17,18]. Several biological substances, including allergens such as house dust mites and some molds, contain molecules that have been shown to trigger Th2 immune responses by activation of dendritic cells. Recently, it has been demonstrated that components of peanut protein also have these adjuvant properties, that is, they enhance the body’s immune response to an antigen. Peanut proteins induce the production of retinoic acid from myeloid dendritic cells, which in turn, promotes TH2 differentiation and gut homing in naive CD4+ T cells. Therefore, exposure to peanut, and potentially other food allergens, may have a priming effect on the immune system skewing it towards Th2 immune responses. In line with the dual allergen hypothesis, exposure to peanut through the skin, as opposed to the oral route which by default promotes oral tolerance, may also promote skewing of the immune system to the allergic phenotype [19]. Several studies have therefore attempted to prevent food allergy by preventing eczema in early life, by reducing the likelihood of sensitization occurring through a damaged skin barrier. Early small studies showed promising reductions in eczema with regular use of emollients starting in early infancy. Unfortunately, recent larger RCTs testing the effectiveness of regular application of skin emollients to improve the skin barrier, have not proven effective in reducing eczema or food allergy [20,21,22]. This may be because the type of skin interventions, or compliance, were not sufficient to maintain the skin barrier, and further RCTs are underway [23,24].

Given that sensitization to aeroallergens can occur via exposure to the respiratory route, it is plausible that sensitization to foods can also occur this way [25]. Recently, evidence has emerged which supports this. In a mouse model, sensitization to peanut has been induced through the airways. Importantly, this required an adjuvant of house dust exposure, which has been shown to contain immunostimulatory agents; exposure to peanut extract or house dust alone was not sufficient to induce sensitization [26]. In another mouse model, peanut sensitization and reaction was induced following intranasal exposure to 100 ug of peanut flour twice per week for 4 weeks, without the need for adjuvant. This response was mediated by follicular helper T cells, which produced IL-4 and IL-21 and promoted production of peanut sIgE [27]. This suggests that the dual allergen exposure hypothesis may extend to the respiratory route. Future investigations of the role that the physical environment plays in the development of food allergy should therefore consider the impact the environment has on both the skin and respiratory epithelium.

### 2.2. Vitamin D

The role of vitamin D in the development of food allergy remains under investigation. Several mechanisms plausibly link vitamin D to food allergy. Vitamin D acts through diverse immunological pathways, some of which are associated with tolerogenic immune functions. For example, vitamin D suppresses excess inflammation through inhibition of pro-inflammatory cytokines and inhibits maturation of dendritic cells, and because immature dendritic cells are tolerogenic, this contributes to T cell tolerance. Vitamin D is also involved in inducing regulatory T cells and inhibiting T cell proliferation associated with the Th2 allergic phenotype [28,29]. Additionally, vitamin D promotes expression of genes encoding proteins necessary for epithelial tight junctions and these are important for maintaining epithelial barrier integrity in both the skin and gastrointestinal tract [28,29].

Sunlight is the main source of vitamin D, although small amounts are present in some foods, such as eggs, and is also fortified in the food supply chain in some countries. Upon reaching the skin, solar ultraviolet radiation band B (UVB) interacts with 7-dehydrocholesterol which is photochemically converted to 25-hydroxy vitamin-D3 [30]. Several environmental factors contribute to how much UVB reaches the skin, including surface reflectance, atmospheric effects such as ozone, cloud cover, the angle of the sun, which is determined by the time of day, season and latitude, and shade from buildings and tree coverage [31,32].

Several factors that are proxies for vitamin D levels have been associated with the risk of food allergy. A strong latitude and food allergy association has been reported with countries further from the equator with lower ambient ultraviolet radiation reporting higher rates of food allergy, adrenaline-autoinjector prescriptions and hospital admissions for food-related anaphylaxis [33,34,35,36,37]. Furthermore, season of birth has been associated with an increased risk of food allergy and adrenaline-autoinjector prescriptions in some studies [38,39,40].

In the Australian HealthNuts study, vitamin D insufficiency (<50 nmol/L) at age 12 months was associated with an 11-fold increase in peanut allergy (adjusted OR, 11.51; 95% CI, 2.01–65.79) and nearly 4-fold increase in egg allergy (adjusted OR 3.79; 95% CI, 1.19–12.08) among infants of Australian-born parents [41]. However, systematic reviews have failed to find consistent evidence of an association for vitamin D insufficiency or vitamin D supplementation and the risk of allergic disease, although comparisons are marred by heterogeneity of studies [42,43]. Furthermore, there is some evidence to support a “U-shaped” association where both high and low vitamin D levels in cord blood in the same population demonstrated an increased risk of allergic outcomes [44,45]. Due to the ambiguity of these results, further RCTs are needed to understand the potential of vitamin D supplementation as a preventative intervention for food allergy and a large randomized controlled trial is currently underway investigating this [46].

## 3. Environmental Risk Factors for Food Allergy

### 3.1. The Microbial Exposure and Biodiversity Hypotheses

The interrelated microbial exposure and biodiversity hypotheses are premised on similar mechanisms. The microbial exposure hypothesis proposes that an absence of exposure to microbes and infections in early life impacts the developing immune system, predisposing to allergic disease. Likewise, the biodiversity hypothesis proposes that exposure to a variety of biodiverse environments, which would subsequently increase exposure to a diversity of microbes, similarly regulates the human gut microbiome and promotes healthy immune system development [47,48]. Commensal microbiota that colonize the skin and gastrointestinal tract influence the maturation of immune responses. Aberrance of the microbial environment can skew the immune system towards the Th2 dependent phenotype which promotes production of IgE antibodies [11,49,50]. Studies have shown that children with food allergy exhibit altered gut microbial composition compared to children without food allergy [51,52].

These hypotheses are supported by a growing body of evidence [50]. Children who are raised on farms and have frequent contact with animals are less likely to develop allergic disease compared to children raised in cities [50]. The prevalence of food sensitization and food allergy is lower in children living in rural areas of South Africa compared to urban areas [5]. Children with older siblings and those who attend childcare in early life or are exposed to pet dogs are also less likely to develop food allergies [50,53]. One study also reported that cleaning of infant pacifiers by parents sucking on it, as opposed to rinsing it in tap or boiling water, was associated with a reduced risk of developing childhood allergic diseases. In this study, the infant’s oral microbiota also differed between the two cleaning methods which suggests that the development of allergy was related to immune system modulation from microbes transferred from the parent’s saliva to the infant [54]. In a more recent study, pacifier use at 6 months of age was associated with challenge-confirmed food allergy at age 12 months and this association was driven by pacifier-antiseptic use (aOR 4.83; 95% C, 1.10–21.18 for infants with use of antiseptics to clean pacifiers, compared with no pacifier use). When the analysis was restricted to only infants who used pacifiers, antiseptic cleaning was still associated with food allergy (aOR, 3.56, 95% CI 1.18–10.77 compared with no antiseptic use) [55].

### 3.2. Air Pollution

Industrialization has increased over recent decades, exposing populations to increased air pollution from cars, diesel exhaust, and bushfires which results in increased air pollutants such as particulate matter (PM) and nitrous oxide (NO). Although there is a large body of evidence linking air pollution to allergic respiratory disease, including asthma prevalence as well as hospitalizations [56], the data for food allergy is sparse.

Several mechanisms plausibly link air pollution to allergic disease pathways. Epithelial cells, which are found in the skin, gastrointestinal tract, and respiratory tract, are the first line of defense against external pathogens and stimuli, including air pollution. These are essential elements of tissue barrier and innate immune responses. When epithelial cells are exposed to external pathogens including pollutants, cytokines are produced and an inflammatory response ensues which can include activation of dendritic cells, Th2 cells, and mast cells. Additionally, this inflammatory response can cause the epithelial barrier’s tight junctions to open which can allow external substances, including allergens, to enter the deeper tissues and blood stream [57,58]. The epithelial barrier plays an important role in allergy development and disruption to the epithelial barrier underpins the dual allergen exposure hypothesis. Multiple factors can disrupt the epithelial barrier including exposure to air pollution, chemicals in detergents as well as genetic mutations, which skew the immune system to Th2 response that underlies allergic sensitization and disease [57].

As part of the European collaborative European Study of Cohorts for Air Pollution Effects, a meta-analysis was performed using data from five European birth cohorts to estimate the effect of ambient air pollution (PM_2.5_, PM_10_, and NO_2_) on the development of allergic sensitization in children up to 8–10 years of age. There was little consistent evidence of an association between air pollution at birth or at the time of outcome measurement on the risk of sensitization to common food and aeroallergens when examined as a combined outcome, at age 4–6 and 8–10 years. When sensitization to food allergens was considered as a separate outcome, the pooled estimate showed increased risk estimates, particularly for current PM_2.5_ exposure and food sensitization at 8–10 years [59].

Data from four European birth cohorts participating in the Mechanisms of the Development of ALLergy (MeDALL) consortium (*n* = 6163 children up to 16 years of age) were combined to assess the relationship between air pollution and sensitization using standardized exposure assessment. Overall, there was no clear evidence of an association between air pollution at birth or time of bio-sampling, and the risk of developing IgE sensitization to common inhalant or food allergens. In the PIAMA cohort, there was consistent evidence that increased air pollution was associated with an increased risk of food sensitization, and although there was a trend of higher risk estimates for food allergens in the other studies, this association was not maintained when the four cohorts were combined in a meta-analysis [19]. These differences may reflect differences in how air pollution was measured, which foods were measured, as well at the impact of other environmental factors that were not measured. Individual analyses conducted in the PIAMA cohort (Netherlands) in a separate publication, found that long-term exposure to air pollution was associated with an increased risk of food sensitization at age 4 years, however, it should be noted that only children with higher familial risk of allergy had specific IgE measured (PM_2.5_, aOR 1.75 (95%CI 1.23–2.47, soot aOR 1.64 (95% CI 1.21–2.23) and NO_2_ aOR 1.49 (95% CI 1.13–1.97)) [60].

Further individual studies found no association between air pollution and risk of food sensitization [61,62]. A limitation of these studies is that air pollution was measured at the home address, which does not account for exposure that occurs away from the home such as childcare and school, where children can spend substantial amounts of time. Additionally, these locations tend to involve outdoor play and exertion which increases susceptibility and exposure to air pollution.

Although there is some evidence, albeit conflicting, that increased exposure to air pollution is associated with an increased risk of food sensitization, its relevance to clinical food allergy is unclear. As IgE sensitization also occurs in children who are able to tolerate the food without reaction, IgE sensitization is a poor proxy for food allergy and known to overestimate the true prevalence of food allergy. To date, no study has examined the association between outdoor air pollution and the risk of food allergy, therefore, further research is needed.

### 3.3. Environmental Greenness

There is a growing concern that the expansion of urban and metropolitan areas will result in a reduction of natural green environments. Environmental greenness encompasses tree, shrubs, and grasses, and provides opportunities to interact with vegetative diversity that in turn supports diversity of fauna and microbes. Environmental green spaces are critical for not only encouraging active lifestyles [63], but for providing people with an opportunity to interact with diverse microbes, vegetation, soil, and pollen [64,65]. Natural green environments play a critical role in promoting healthy lifestyles and are associated with benefits to mental health, cardiovascular disease, immune health, and reducing all-cause mortality [37,38,66,67,68]. The biodiversity hypothesis is one pathway through which environmental greenness may promote human, and in particular immune health [48,69]. In addition, environmental greenness interacts with other features of the environment and can influence health by reducing the harmful effects of other environmental factors such as air pollution or extreme heat. While studies have examined the association between environmental greenness and the development of other allergic diseases, few have examined food sensitization and allergies.

Only one study of 631 children up to age 15 years old in Germany has examined the association between residential greenness and food sensitization. This study found little evidence of an association between residential greenness and food sensitization but did find that increased exposure to allergenic trees was associated with an increased risk of food sensitization (aOR 1.59 95% CI 1.05–2.42) [70]. A possible explanation for this finding is cross-reactivity between some pollen and peanut allergens; cross-reactivity is where IgE antibodies specific to one allergen recognize, bind to and induce an immune response to other similar allergenic molecules [71,72]. The HealthNuts study, to date, is the only study to examine the association between environmental greenness and the risk of challenge-confirmed food allergy. Increased exposure to environmental greenness, measured by the Normalized Difference Vegetation Index, was associated with an increased risk of peanut and egg allergy [73].

As food allergy is one of several atopic diseases which often co-occur and share pathophysiological features, evidence from other allergic disease research may be informative. A systematic review of 11 studies on greenspace and aeroallergen sensitization found mixed evidence of associations [74]. The evidence regarding other allergic diseases, such as asthma and allergic rhinitis, is also mixed. Some studies have shown that increased greenness has a protective effect against developing asthma [65,75] and allergic rhinitis [76,77]. In contrast, other studies have shown an increased risk for the same outcomes [78,79,80].

Further research on the role of environmental greenness and risk of developing allergies is needed. Living in a greener environment does not necessarily equate to interacting with the natural environment, therefore, sensitive measures that capture specific types of vegetation, time spent outdoors in natural environments, as well as consideration of interactions with other environmental features such as air pollution, may help clarify previous inconsistent findings.

### 3.4. Pollen

Pollen can impact the immune system in several ways that are relevant to allergic pathways, skewing the developing immune system to the Th2-dependent allergic phenotype. Aqueous pollen extract proteins and pollen lipids modulate dendritic cell function and stimulate Th2 chemokine production, characteristic of the allergic response. Additionally, some pollen proteases can damage epithelial barrier function in airways which may increase uptake of pollen antigens [81]. Based on recent evidence that peanut sensitization may occur through respiratory exposure to peanut allergens, high pollen levels may facilitate this by damaging the epithelial barrier and increasing the potential for food sensitization [25].

Directly measured grass pollen exposure during several periods of pregnancy and the first year of life was associated with a moderate increase in risk of food sensitization in 12-month-old infants in the Australian HealthNuts cohort. Risk of challenge-confirmed peanut allergy was also increased but only among infants with a maternal history of food allergy [82]. Cross-reactivity has been recognized between some food and pollen allergens, for example, some peanut allergens have known cross-reactivity to birch and plane tree pollen allergen [83,84]. It is possible that increased exposure to environmental allergens such as grass or pollen allergens through the respiratory or cutaneous route may prime the immune system to be more reactive to these protein families that are shared with food allergens.

## 4. Interplay of Environmental Factors

**Figure 1:**
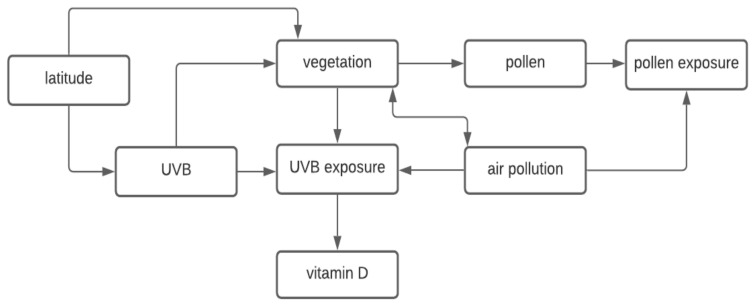
The interplay between environmental factors that may be relevant for food allergy development.

Investigation of the relationship between the outdoor environment and risk of food allergy is complicated by the complex interplay between the different environmental factors. For instance, the four environmental factors we have described here (vitamin D, air pollution, greenness, and pollen) are interrelated with Figure 1 illustrating these interrelationships. The human body synthesizes vitamin D after being exposed to UVB radiation, which varies by latitude [32]. Vegetation (i.e., greenness) is also related to latitude, in part due to dependence and variation of solar radiation exposure. Greener areas with more vegetation may encourage people to spend more time outdoors and therefore have greater opportunity to be exposed to UVB radiation which enables vitamin D synthesis, and pollens or molds, depending on the timing and type of greenness [85]. Yet, on the other hand, sufficiently high levels of vegetation could also reduce UVB radiation exposure as the tree canopy can block out sunlight. Diversity of environmental microbiota is also related to vegetation levels [86,87]. Pollen requires the presence of vegetation and therefore, vegetation can determine levels of pollen exposure. It appears that pollen may also relate to air pollution, with pollutants potentially facilitating pollen allergen release, stimulating IgE-mediated responses, and increasing pollen allergenic potential [88,89,90]. Finally, there is a complex interrelationship between air pollution and vegetation since vegetation may mitigate or increase air pollutant exposures depending on the location and structure of the vegetation and air pollution can have detrimental effects on vegetation [91,92].

There is also complexity underlying the development of allergy which is likely due to the interplay between multiple influences such as exposure to air pollution and chemicals, inflammation and infection, alterations to the microbiome, food allergen exposure, and genetic predispositions [57]. For example, although there is evidence from animal models to support peanut sensitization through the respiratory route, with or without adjuvants, and Kulis et al. propose that interrelationships with other environmental factors, such as air pollution, in addition to dust, may play a role in promoting airway sensitization. This may be one explanation for why there is a higher prevalence of food allergy in urban versus rural environments [25]. These complexities suggest that a systems approach, which considers the interrelationships between multiple environmental factors, may be important in unraveling the relationship between environmental factors and food allergy specifically [57], and human health in general [47,48,93,94].

## 5. Conclusions

In conclusion, the development of food allergy is multifactorial, and there is a complex interplay between environmental factors that may contribute to the risk of food allergy. While there is strong evidence to support the early introduction of allergenic solids to reduce the risk of food allergy, early introduction alone is unlikely to be sufficient to prevent all food allergy. The dual allergen exposure hypothesis is supported by a growing body of evidence, with recent studies proposing that this hypothesis may include the respiratory epithelial barrier in addition to the skin. Plausible mechanisms that could explain a role for vitamin D, air pollution, environmental greenness, and pollen exposure in the development of food allergy exist, however, evidence to date is conflicting or insufficient as few studies have examined these environmental factors with objective measures of IgE-mediated food allergy. Future research needs to consider the interrelationships between multiple environmental factors.

## Data Availability

Not applicable.
